# Tobacco Use Among Youths — Argentina, 2007 and 2012

**Published:** 2014-07-11

**Authors:** Jonatan Konfino, Daniel Ferrante, Lucila Goldberg, Roberta Caixeta, Krishna Mohan Palipudi

**Affiliations:** 1 Argentina Ministry of Health; 2 Pan American Health Organization, Washington, DC; 3 Office of Smoking and Health, National Center for Chronic Disease Prevention and Health Promotion, CDC

Tobacco use is the leading preventable cause of deaths worldwide (1). The MPOWER package, the six recommended policies of the World Health Organization (WHO) to reverse the tobacco epidemic, strongly recommends monitoring tobacco use trends (2). Because evidence indicates that smoking addiction often starts before the age of 18 years (3), there is a need to monitor tobacco use among youths. During 2011, a National Tobacco Control Law was enacted in Argentina that included implementation of 100% smoke-free environments, a comprehensive advertising ban (prohibiting advertising, promotion, and sponsorship of cigarettes or tobacco products through any media or communications outlets), pictorial health warnings, and a prohibition against the sale of tobacco products through any means to persons aged <18 years (4,5). To ascertain trends in tobacco use among youths in Argentina, the Argentina Ministry of Health and CDC analyzed data from the Global Youth Tobacco Survey (GYTS) for 2007 and 2012 (the next year that it was administered in Argentina). The findings indicated that the overall proportion of youths aged approximately 13–15 years who reported ever smoking a cigarette declined from 52.0% in 2007 to 41.9% in 2012 with significant decreases among both males and females. In 2012, 52.5% of youths in Argentina reported secondhand smoke (SHS) exposure in their homes and 47.5% in enclosed public places in the 7 days preceding the survey. Increased public education and tobacco control efforts will be important to discouraging tobacco use and decreasing SHS exposure among youths in Argentina.

GYTS is a nationally representative, school-based survey of students in grades associated with the ages of 13–15 years and is designed to produce cross-sectional estimates for each country. GYTS uses standardized sample design, core questionnaire, and data collection procedures. The survey assists countries in fulfilling their obligations under the WHO Framework Convention on Tobacco Control to generate comparable data within and among countries. In Argentina, GYTS was conducted at a national level for the first time in 2007 (6) and repeated in 2012, under the coordination of the Ministry of Health. In 2007, the survey was completed by 4,926 students aged 13–15 years, with an overall response rate of 68.2%. In 2012, the survey was completed by 2,069 students aged 13–15 years, with an overall response rate of 76.9%.

GYTS uses a two-stage sample design with schools selected with the probability of selection proportional to school enrollment size. The classrooms within selected schools are chosen randomly, and all students in selected classes are invited to participate in the survey (7). The survey uses a globally standardized core questionnaire with a set of optional questions about tobacco use and key tobacco control indicators, which permit adaptation to meet the needs of the country. The questionnaire covers the following topics: tobacco use (smoking and smokeless), cessation, exposure to SHS, protobacco and antitobacco media and advertising, access to and ability to obtain tobacco products, and knowledge and attitudes about tobacco. The questionnaire is self-administered, uses scannable answer sheets, and is anonymous to ensure confidentiality.

For this report, statistical software was used to analyze weighted 2007 and 2012 Argentina GYTS data for the following categories: current cigarette smoker, frequent cigarette smoker, current smoker of other tobacco, ever smoked a cigarette, current smokeless tobacco user, current tobacco user, and exposure to SHS. Differences between rates were determined to be statistically significant by Student’s t-test. The overall current cigarette smoker rate (the weighted percentage of respondents who reported having smoked a cigarette any time during the previous 30 days) was 24.5% in 2007 and 19.6% in 2012 (Table). Among females, the rates were 27.3% and 21.5%, respectively; among males, the rates were 21.1% and 17.4%, respectively. Rates of ever cigarette smoking (ever taking a puff on a cigarette) decreased from 52.0% in 2007 to 41.9% in 2012. Among females, the rate decreased from 54.8% to 43.3%; among males, the rate decreased from 48.9% to 40.5%.

The overall smokeless tobacco user rate was 4.3% in 2007 and 3.7% in 2012, a decrease that was not statistically significant, and remained higher among males than females (Table). The tobacco use rate (either smoked or smokeless) also showed a decrease that was not statistically significant, from 28.0% to 24.1%, and remained higher among females than males. In addition, the overall rates for frequent smoking (smoking on 20 or more days of the previous 30 days) were 5.6% in 2007 and 4.1% in 2012, and remained higher among females.

Exposure to SHS in enclosed public places decreased from 54.7% in 2007 to 47.5% in 2012 (Table). In 2012, 50.1% of females and 44.5% of males reported SHS exposure in enclosed public places. Rates were higher in 2012 for exposure to SHS inside the home than in enclosed public places: 52.5% overall, 55.7% among females, and 48.8% among males.

## Discussion

The findings in this report show a decrease in current use of tobacco among adolescents in Argentina. Although the current use rates for both males and females were lower in 2012 than in 2007, cigarette smoking rates (one or more cigarettes in the past 30 days) remained at approximately 20%; without further prevention efforts these rates will result in avoidable tobacco-related morbidity and mortality in this generation. Although the differences were not statistically significant, the prevalence of frequent cigarette smoking likely was higher among females (5.0%) when compared with males (3.1%), and the prevalence of current smokeless tobacco use (any use in the previous 30 days) likely was higher among males (4.4%) when compared with females (3.0%). These findings suggest that sex-specific tobacco control approaches among youths might merit consideration in Argentina. In addition, the observation that youths had high exposure rates to SHS in enclosed public places, similar to what has been reported in other regions (8), shows the challenge of protecting youths from public SHS. Finally, over half of youths in Argentina reported exposure to SHS in their homes, suggesting the importance of public education regarding the dangers of SHS exposure.

The findings in this report are subject to at least four limitations. First, because GYTS is limited to students, the survey is not representative of all youths aged 13–15 years. However, in Argentina as in most countries, the majority of persons in this age group attend public, private, or technical schools (9). Second, these data apply only to youths who were in school on the day of the survey and who completed the survey. Third, the survey response rates were 68.1% and 76.9% in 2007 and 2012, respectively, and nonresponse bias might have affected the results. Finally, data were based on the self-report of students, who might underreport or overreport their behaviors. The extent of this bias cannot be determined from these data; however, reliability studies in the United States have indicated good test/retest results for similar tobacco-related questions (10).

The findings that “ever tobacco” use among adolescent males and females is decreasing in Argentina are consistent with the decrease reported among adults in Argentina in the 2012 Global Adult Tobacco Survey.* Although Argentina has not ratified the WHO Framework Convention on Tobacco Control, these findings could be partly related to the country’s national tobacco control law in 2011 and to the work that has been done at a subnational level over more than a decade to create smoke-free environments.

What is already known on this topic?

Argentina has implemented work at the subnational level regarding smoke-free policies for more than a decade. The country conducted the Global Youth Tobacco Survey (GYTS) at a national level for the first time in 2007. The GYTS is a standardized, nationally representative, school-based survey of students aged approximately 13–15 years.

What is added by this report?

In 2012, Argentina repeated GYTS for the first time since 2007. The proportion of respondents who reported that they had ever puffed on a cigarette declined from 52.0% in 2007 to 41.9% in 2012. Frequent smoking (smoking ≥20 days in the previous 30 days) in 2012 was reported more commonly by females (5.0%) than males (3.1%), but was lower than in 2007 (6.0% and 4.9%, respectively). Current smokeless tobacco use was more commonly reported by males (4.4%) than females (3.0%). Secondhand smoke exposure was reported by a majority (52.5%) of students in their homes and by 47.5% in enclosed public places in the 7 days preceding the survey.

What are the implications for public health practice?

Despite progress, tobacco use remains a threat to the health of youths in Argentina. In 2012, approximately one fifth of youths aged 13–15 years were current smokers, and nearly half were exposed to secondhand smoke in enclosed public places. Efforts to reduce secondhand smoke and discourage tobacco use among youths are needed, and different approaches for females and males might be appropriate.

WHO’s MPOWER framework provides evidence-based interventions that countries can use to reduce tobacco use. Although Argentina has made progress on key components of MPOWER, including surveillance as evidenced by these data, many opportunities for prevention exist. For example, because price increases have been shown to be the most effective single measure to decrease consumption and discourage initiation among youths (3), increasing tobacco product prices might be an effective approach to promote further decline in youth smoking in Argentina. In addition, increased enforcement of the current national law could help to address the problem, including preventing access by youths to tobacco products, restricting advertising at point of sale, and protecting youths from SHS.

## Figures and Tables

**TABLE:** Prevalence of tobacco use and exposure to secondhand smoke (SHS) among youths aged 13–15 years — Global Youth Tobacco Survey, Argentina, 2007 and 2012

	2007			2012			Overall percentage point change from 2007 to 2012	p-value*
		
	Overall	Males	Females	Overall	Males	Females		
						
	% (95% CI)			% (95% CI)				
**Smoked tobacco use**								
Current cigarette smokers†	24.5 (22.2–27.0)	21.1 (18.5–23.8)	27.3 (23.4–31.6)	19.6 (16.4–23.3)	17.4 (14.7–20.5)	21.5 (17.1–26.7)	−4.9	0.021
Frequent cigarette smokers§	5.6 (4.2–7.4)	4.9 (3.8–6.4)	6.0 (3.7–9.5)	4.1 (3.0–5.6)	3.1 (2.1–4.6)	5.0 (3.3–7.3)	−1.5	0.158
Current smokers of other tobacco¶	6.7 (5.6.8.1)	9.1 (7.4–11.2)	4.6 (3.5–6.2)	6.5 (5.0–8.3)	7.0 (5.4–9.1)	6.0 (4.3–8.2)	−0.2	0.810
Ever cigarette smokers**	52.0 (49.5–54.5)	48.9 (45.6–52.2)	54.8 (50.6–58.9)	41.9 (38.2–45.8)	40.5 (36.5–44.6)	43.3 (38.2–48.6)	−10.1	<0.001
**Smokeless tobacco use**								
Current smokeless tobacco users††	4.3 (3.5–5.2)	5.5 (4.3–6.9)	3.2 (2.4–4.3)	3.7 (2.9–4.8)	4.4 (3.2–6.1)	3.0 (2.0–4.5)	−0.6	0.420
**Tobacco use**								
Current tobacco users§§	28.0 (25.9–30.3)	26.1 (23.6–28.8)	29.7 (25.7–34.0)	24.1 (20.8–27.8)	22.7 (20.1–25.5)	25.4 (20.3–31.2)	−3.9	0.058
**Exposure to SHS**								
Exposure to SHS inside any enclosed public place	54.7 (51.9–57.4)	51.7 (48.3–55.2)	57.7 (54.4–61.0)	47.5 (43.1–51.9)	44.5 (39.7–49.4)	50.1 (44.6–55.7)	−13.6	0.006
Exposure to SHS inside the home	NA	NA	NA	52.5 (49.8–55.1)	48.8 (45.0–52.7)	55.7 (52.3–59.1)	NA	NA

**Abbreviations:** CI = confidence interval; NA = not available.

* Comparing overall values for 2007 and 2012.

† Smoked cigarettes any time during the preceding 30 days.

§ Smoked cigarettes on 20 or more of the preceding 30 days.

¶ Smoked tobacco other than cigarettes anytime during the preceding 30 days.

** Ever smoked cigarettes, even one or two puffs.

†† Used smokeless tobacco anytime during the preceding 30 days.

§§ Used smoked tobacco and/or smokeless tobacco anytime during the preceding 30 days.

## Footnotes

* Global Adult Tobacco Survey Argentina 2012 [Spanish]. Available at http://www.msal.gov.ar/ent/images/stories/vigilancia/pdf/GATS_FactSheetARG_FINAL.pdf.
